# Management of cryptoglandular fistula-in-ano among gastrointestinal surgeons in the Netherlands

**DOI:** 10.1007/s10151-021-02446-3

**Published:** 2021-04-15

**Authors:** L. Dekker, D. D. E. Zimmerman, R. M. Smeenk, R. Schouten, I. J. M. Han-Geurts

**Affiliations:** 1 Department of Surgery, Amsterdam University Medical Center, Location AMC, Meibergdreef 9, 1105 AZ Amsterdam, The Netherlands; 2 Department of Surgery, Proctos Kliniek, Bilthoven, The Netherlands; 3 Department of Surgery, Elisabeth-TweeSteden Ziekenhuis, Tilburg, The Netherlands; 4 Department of Surgery, Albert Schweitzer Ziekenhuis, Dordrecht, The Netherlands; 5 Department of Surgery, Flevoziekenhuis, Almere, The Netherlands

**Keywords:** Anal fistula, Survey, Surgical, Therapy, Diagnostic, Perianal fistula, Fistula in ano

## Abstract

**Background:**

Management of cryptoglandular fistula-in-ano (FIA) can be challenging. Despite Dutch and international guidelines determining optimal therapy is still quite difficult. The aim of this study was to report current practices in the management of cryptoglandular FIA among gastrointestinal surgeons in the Netherlands.

**Methods:**

Dutch surgeons and residents who are treating FIA regularly were sent a survey invitation by email. The survey was available online from September 19 to December 1 2019. The questionnaire consisted of 28 questions concerning diagnostic and surgical techniques in the treatment of intersphincteric and transsphincteric FIA.

**Results:**

In total, 147 (43%) surgeons responded and completed the survey. Magnetic resonance imaging was the preferred diagnostic imaging modality (97%) followed by the endo-anal ultrasound (12%). In case of a high FIA, 86% used a non-cutting seton. Most respondents removed a seton between 6 weeks and 3 months (*n* = 84, 58%). Fistulotomy was the procedure of preference in low transsphincteric (86%) and low intersphincteric FIA (92%). Mucosal advancement flap (MAF) and ligation of intersphincteric fistula tract (LIFT), with 78% and 46%, respectively, were the procedures that were applied most often in high transsphincteric FIA. In high intersphincteric FIA 67% performed a MAF and 33% a fistulotomy. Thirty-three percent of all respondents stated that they habitually closed the internal fistula opening, half of them used a Z-plasty. For debridement of the fistula tract the preferred method was curettage (78%).

**Conclusions:**

Dutch gastrointestinal surgeons use various techniques in the management of FIA. Novel promising techniques should be investigated adequately in sufficient large trials to increase consensus. A core outcome measurement and a prospective international database would help in comparing results. Until then, treatment should be adjusted to the individual patient, governed by fistula characteristics and patient choice.

## Introduction

Fistula-in-ano (FIA) has been challenging to manage for thousands of years. Hippocrates was the first who described and analyzed the etiology and technique of healing this troublesome benign disease [1, 2]. Yet, therapy for FIA has not fundamentally changed. Therapy is aimed at closure of the fistula and symptom relief whilst minimizing functional impairment. Despite current Dutch and international guidelines, determining optimal therapy is still quite difficult in the individual patient. A probable cause is the scarce evidence regarding the best practice in treating FIA [3–6]. This concerns all areas of management: diagnostics, operative treatment, follow-up and treatment of recurrent disease.

Ideally, surgical management aims to heal fistula with preservation of fecal continence. Simple FIA can be safely treated by fistulotomy (lay open) with high healing rates between 80–100% [7–9]. Complex fistulas are more challenging for the surgeon due to the higher risk of fecal incontinence and recurrence [10, 11]. These fistulas are often treated by seton placement prior to subsequent sphincter-preserving surgery. Sphincter-preserving techniques include mucosal advancement flap (MAF) with reported healing rates between 70–80% [12, 13], and ligation of the intersphincter fistula tract (LIFT) with a reported healing rate of 69% for cryptoglandular FIA [14–16]. Other sphincter-sparing procedures that have been developed are: tissue-adhesive and biomaterials, stem cells, fistula laser closure (FiLaC™), video-assisted anal fistula treatment (VAAFT) and over-the-scope clip (OTSC®). Some of these procedures have been quickly adopted, without a prior pilot or implementation study. Also, technical variations of procedures are performed in an attempt to improve outcome [11, 17–19].

The question still remains, which procedure leads to optimal outcome for the individual patient suffering from FIA? Many studies have attempted to answer this question by comparing techniques through evaluating outcome measurements such as fecal incontinence, recurrence and/or fistula closure. Data are often difficult to compare due to heterogeneity between studies. For that reason, a core outcome set (COS) for perianal fistula is currently under development including patient-related items [20].

Our objective was to assess the contemporary approach in surgical management of cryptoglandular FIA in the Netherlands and to determine whether current management follows current guidelines.

## Materials and methods

### Design of the survey

The survey consisted of 28 questions, formulated by two authors (IH and LD). To compare our results with the management of cryptoglandular FIA worldwide, the questions were partially based upon the international survey developed by Ratto et al. [21]. The questions were reviewed by three co-authors (gastrointestinal- and colorectal surgeons) after which the survey was edited and co-authors conducted a pilot for testing validity.

The survey consisted of topics concerning baseline characteristics such as respondents function, sex, workload, type of hospital, years of experience in management of cryptoglandular FIA and number of cases treated per year. Seton use was assessed by questions covering material and duration. Other questions assessed diagnostic techniques, surgical approach, (not) dealing with an internal opening and expertise with the different surgical approaches. If the question mentioned ‘high intersphincteric’ FIA, it was generally described as a intersphincteric FIA with a high internal opening. The survey was in Dutch and was created using a web-based program called Survey Monkey. Ten questions were multiple-choice and 18 were single-answer questions (Appendix 1 the English translation is provided in Appendix 1). It was explicitly stated in the invitation that all questions were related to cryptoglandular fistulas only.

The survey was sent by email to all members of the Dutch Working Group Coloproctology as well as to all gastrointestinal- and colorectal surgeons, fellows and residents of each hospital in the Netherlands treating FIA regularly. Data were checked by calling the local secretariats. Contact information was retrieved from the Dutch Association for Surgery. One email reminder was sent during the period of online availability of the survey. A link to the survey was disseminated via LinkedIn and via the newsletter of the Dutch Workgroup Coloproctology as a reminder. The survey was available online from September 19 to December 1 2019. As this study did not apply the Medical Research Involving Human Subjects Act (WMO), approval by the ethics committee was not required.

### Data analysis

To prevent missing data, all questions were mandatory with automated skip logic. The web-based program automatically collected all data after which the data were exported to a Microsoft Excel spreadsheet and then imported to SPSS. Descriptive analyses were performed on all data. Categorical outcome data across groups were analysed using the Chi-square test. IBM SPSS version 25 was used.

## Results

### Respondents’ characteristics

In total, 342 invitations were sent by email to gastrointestinal surgeons, fellows and residents. Four email addresses with an invalid domain were excluded. One hundred and forty-six respondents (43%) completed the survey, 117 by answering the email invitation and 29 using the web link*.* Respondents’ characteristics are shown in Table 1. Most respondents (52%) had more than 10 years of experience with treating FIA. Only 33% performed more than 30 procedures per year. Patients who had their first appointment in the outpatient clinic were mostly counseled by a surgeon or resident. Overall, no significant differences in management were seen regarding experience in number of surgical procedures performed per year.

**Table 1:** Respondents characteristics

	*N *(%)
Sex	
Male	103 (71)
Female	42 (29)
Specialty	
Gastrointestinal surgeon	108 (75)
General surgeon	12 (8)
Fellow	6 (4)
Resident (in training)	19 (13)
Work load	
Fulltime	113 (78)
Part-time	32 (22)
Type of hospital	
Academic	14 (10)
Non-academic (peripheral)	124 (86)
(Private) clinic	7 (5)
First visit contact outpatient clinic (mc)	
Surgeon	142 (98)
Fellow	51 (35)
Resident (in training)	74 (51)
Resident (not in training)	26 (18)
PA or nurse practitioner	10 (7)
Experience treating anal fistulas	
1–5 years	35 (24)
5–10 years	34 (23)
10–20 years	51 (35)
>20 years	25 (17)
Experience in total FIA procedures per year	
>50	20 (14)
30–50	27 (19)
10–30	72 (50)
0–10	26 (18)

*mc* multiple choice; *PA?* physician’s assistant; *FIA* fistula-in-ano

### Diagnostic imaging

Table 2 shows the diagnostic imaging modalities used by respondents. Diagnostic imaging was commonly used in case of complex fistulas (*n* = 133, 78%) and recurrent fistulas (*n* = 92, 63%). The respondents who answered ‘always’ (*n* = 19, 13%) were not included. Magnetic resonance imaging (MRI) was used far more often (97%) than endo-anal ultrasound (12%).

**Table 2:** Diagnostic techniques used by respondents

	*N* (%)
Reason for diagnostic imaging (mc)	
Recurrent FIA	92 (63)
Complex FIA	113 (78)
Prior to seton placement	26 (18)
Prior to surgical procedure	49 (34)
Prior to abscess drainage	0 (0)
Always	19 (13)
Type of diagnostic technique (mc)	
MRI	141 (97)
CT scan	0 (0)
Endo-anal ultrasound	18 (12)
No diagnostic technique at all	1 (1)

*mc* multiple choice; *FIA* fistula-in-ano; *MRI* magnetic resonance imaging; *CT* computed tomography

### Seton treatment

The main reason for seton placement was the complexity of the fistula in 112 respondents (77%), followed by the presence of excessive inflammation/suppuration in 67 respondents (46%). Nine percent of respondents indicated to use a seton in all cases whereas only one respondent never uses a seton (Table 3). Silicone was the most commonly used type of seton (68%), followed by the Comfort Drain and SuperSeton® (39% and 13%, respectively), which are characterized by the absence of knots. Fifty-eight percent of the respondents removed the seton between 6 weeks and 3 months, while 19% left it in place until the next surgical procedure.

**Table 3:** Seton treatment by respondents

	*N *(%)
Use of seton placement (mc)	
Always	13 (9)
Purulent FIA	67 (46)
High FIA	112 (77)
Recurrent FIA	51 (35)
Never	2 (1)
Type of seton use (mc)	
Silicone (e.g., vessel loop)	98 (68)
Comfort drain	57 (39)
Surgical thread (e.g., mersilene)	25 (17)
SuperSeton®	19 (13)
Time to remove seton (sa)	
< 6 weeks	1 (1)
Between 6 weeks and 3 months	84 (58)
> 3 months	32 (22)
Until next surgical procedure	28 (19)

*mc* multiple choice; *sa* single answer; *FIA* fistula-in-ano

### Surgical techniques and experience

#### Low fistula-in-ano

**Fig. 1:**
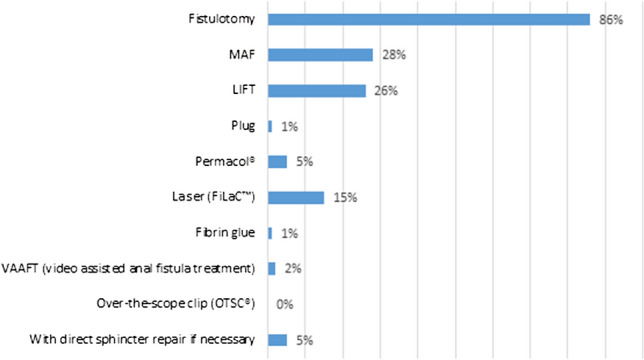
Choice of treatment for low transsphincteric fistula-in-ano (multiple choice). *MAF* mucosal advancement flap; *LIFT* ligation of intersphincteric fistula tract

**Fig. 2:**
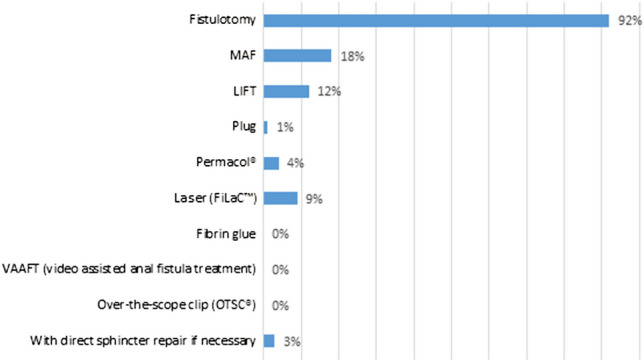
Choice of treatment for low intersphincteric fistula-in-ano (multiple choice). *MAF* mucosal advancement flap; *LIFT* ligation of intersphincteric fistula tract

Figures 1 and 2 illustrates the choice of surgical techniques in low transsphincteric and intersphincteric FIA. Fistulotomy was performed by the majority of the respondents, 86% and 92%, respectively. Still, more than 25% of respondents indicated that they treated low transsphincteric FIA with MAF or LIFT (28% and 26%, respectively). For low intersphincteric FIA, this was 18% by MAF and 12% by LIFT.

**Fig. 3:**
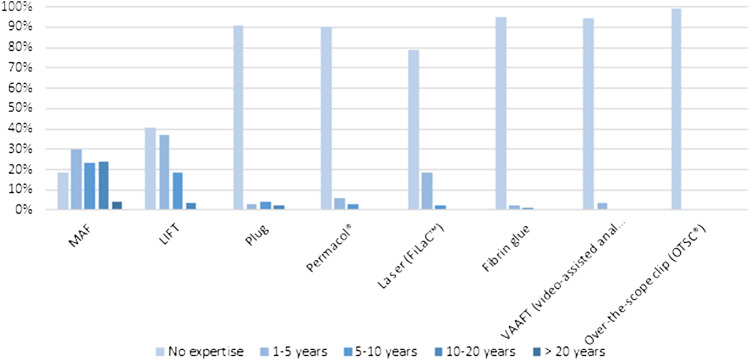
Personal expertise with different techniques. *MAF* mucosal advancement flap; *LIFT* ligation of intersphincteric fistula tract

Eighty-one percent of the respondents had experience with MAF technique, compared to 59% with LIFT (Fig. 3).

#### High fistula-in-ano

**Fig. 4:**
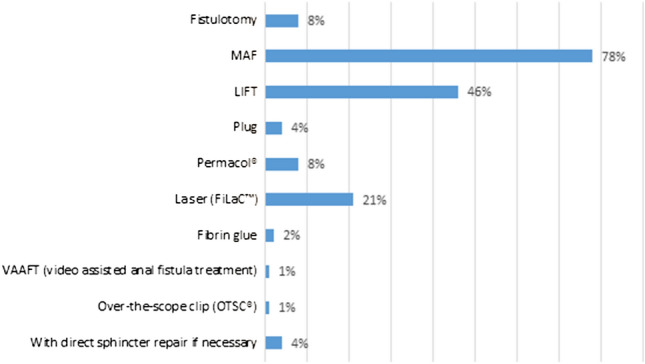
Choice of treatment for high transsphincteric fistula-in-ano (multiple choice). *MAF* mucosal advancement flap; *LIFT* ligation of intersphincteric fistula tract

**Fig. 5:**
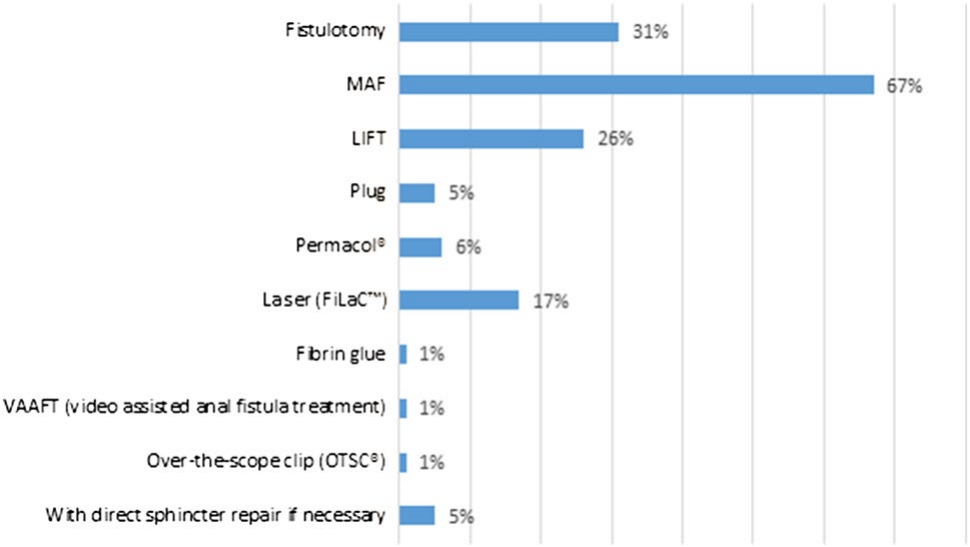
Choice of treatment for high intersphincteric fistula-in-ano (multiple choice). *MAF* mucosal advancement flap; *LIFT* ligation of intersphincteric fistula tract

In case of high transsphincteric FIA, most respondents performed a MAF (78%) or LIFT (46%) (Fig. 4). Twenty-one percent of the respondents treated high transsphincteric fistula with FiLaC™ while almost 80% did not have any experience with this procedure (Fig. 3). The preferred treatment modality for intersphincteric FIA with a high internal opening was more diverse with MAF in first place (67%) followed by fistulotomy (31%) (Fig. 5). LIFT (26%) and FiLaC™ (17%) were also frequently performed in intersphincteric FIA.

Experience with techniques other than MAP and LIFT was limited. Personal experience with plug, Permacol® and fibrin glue was between 5 and 10%. Most respondents had no experience with more novel approaches like VAAFT (94%) and only one respondent had experience with the OTSC®.

### Internal opening

Thirty-three percent of all respondents declared that they closed the internal fistula opening when performing any procedure that allows closure, while 9% never did (Table 4). When performing LIFT 23% indicated that they closed the internal opening. Fifty percent of the respondents who closed the internal fistula opening used a Z-suture and 39% used a normal suture. The remaining 11% closed the internal fistula opening in a different manner. If the internal fistula opening was not found, the majority (66%) did a fistulectomy or core out of the fistula tract, 31% did an excoriation of the fistula tract and 3% did nothing. The preferred method for debridement of 35 (78%) respondents was curettage, 6 (13%) used a brush, 2 (1.4%) used the diathermy needle, 1(0.7%) used a gauze and one a scalpel.

**Table 4:** In what circumstances was the internal fistula opening closed (multiple-choice)

	Total *N* (%)
Always	33 (23)
Never	13 (9)
When performing a MAF	90 (62)
When performing a LIFT	34 (23)
When performing a plug	5 (3)
When performing Permacol	10 (7)
When performing laser	12 (8)
When performing fibrin glue	0 (0)
When performing VAAFT	2 (1)
When performing OTSC®	0 (0)

*MAF* mucosal advancement flap; *LIFT* ligation of intersphincteric fistula tract; *VAAFT* video-assisted fistula treatment; *OTSC* over-the-scope clip

## Discussion

Despite the high prevalence of FIA and a plethora of scientific literature on the subject, there is still no clarity about what is best practice. The present study provides an overview of the current approach in management of FIA amongst gastrointestinal surgeons in the Netherlands.

FIA is most often classified using Parks classification: intersphincteric, transsphincteric, suprasphincteric and extrasphincteric [22]. To aid decision-making in determining the choice of procedure, FIA can be described as high or low, based on the nature of the primary tract. Low fistulas are subcutaneous, intersphincteric or low transsphincteric (involving no more than 1/3 of external anal sphincter), and high fistulas are higher transsphincteric, suprasphincteric or extrasphincteric [23].

Preoperative assessment of anatomy in recurrent and complex anal fistulas by diagnostic imaging has been shown to improve surgical outcome [24] and is therefore recommended in international guidelines [3–6]. Recurrence of perianal fistula is often due to secondary fistula extensions missed during initial surgery. Delineating the fistula pattern prior to surgery with MRI or three-dimensional (3D)-endoanal ultrasound (3D-EAUS) can help to avoid iatrogenic sphincter damage. Both imaging techniques have proven to be superior to examination under anesthesia (EUA) in identifying secondary tracts and identification of the internal orifice [25]. In experienced hands, 3D-EAUS has an excellent sensitivity and specificity in mapping of fistula tracts [26]. Main limitations of 3D-EAUS lie in the identification of pelvirectal abscesses and supralevator tracts. MRI has advantages as reghars soft tissue contrast, operator independence, but has higher costs, a longer execution time and often lower availability. In the cases of complex disease and/or no clear diagnosis at 3D-EAUS, MRI can be a complementary diagnostic tool to previous 3D-EAUS. The majority of the respondents indicated that they used imaging preceding surgery in complex and recurrent fistula. MRI was used far more often than EAUS (97% versus 12%). This is in contrast to the international study by Ratto where a greater proportion of respondents (70%) is familiar with the use of EAUS. It can be assumed that reliance on 3D-EAUS will be higher in hospitals with availability of this device and where surgeons do their own imaging in an outpatient setting as is, to our knowledge, more customary in several European countries. As every corrective procedure for anal fistula has its own specific indications and complications, accurate assessment of a patient’ s anal anatomy and anal fistula by high quality imaging may thus lead to patient tailored advice and treatment.

Setons are frequently used for several reasons. Loose setons are often used for drainage, reducing inflammation and are usually left in place until the acute inflammation has resolved [11]. They are also often used in two-staged surgery preceding a sphincter preserving procedure [27, 28]. There is however no evidence that this leads to better outcome [29–31]. In case there is no intention to perform subsequent surgery, a seton can also be left in situ for an indefinite period of time. There are many different types available made out of diverse materials [32]. It is obvious that efforts to make a seton as comfortable as possible will be much appreciated by the patient. A knot-free seton is proven to be associated with improved quality of life [33]. With 39% of respondents choosing a Comfort Drain and 13% a SuperSeton®, the results of our study suggest that attention is being paid to make wearing a seton more agreeable. It has to be noted however, that also when no commercially produced knotless setons are available, an effort can (and should) be made to make the seton comfortable. Moreover, it should be noted that knotless setons may be more prone to being lost by the patient than knotted setons [34]. The majority of the respondents is accustomed to leaving the seton in situ for a considerable period of time. Fifty-eight percent of the respondents removed the seton between 6 weeks and 3 months. There is no consensus on timing of removal in the literature. The review by Subhas et al., describing variations in materials and techniques in treatment with setons, reports an average duration varying from 14 days till 14 months [32]. Interestingly, what happens to fistulas after loss or removal of a seton without additional surgical therapy is unknown.

The majority of the respondents treated low intersphincteric (86%) and low transsphincteric FIA (92%) with fistulotomy. This data are in line with the literature [35]. Quite a few of the respondents indicated that they perform a MAF or a LIFT procedure in case of low intersphincteric FIA, in contrast to guideline recommendations. It would be interesting to know if this concerns a select patient group, for example female patients with an anteriorly located FIA, or patients with already compromised continence. Although the survey contained questions on low transsfincteric and low intersphincteric FIA, distinguishing between low inter- and low transsfincteric FIA is of dubious importance since it has no consequences for therapy.

Postoperative impaired continence after fistulotomy for low and mid FIA (lower 2/3 of external anal sphincter) is reported in up to 22% of patients [36]. The existing literature suggests there is a positive effect on postoperative continence after fistulotomy and fistulectomy with primary sphincter repair [37–40]. Direct sphincter repair was performed by 3–5% of the respondents in our study. In the international study by Ratto 9–19% performed direct sphincter repair following fistulotomy for intersphincteric and transsphincteric FIA [21]. As Ratto mentioned, this difference could be due to variations across geographic regions. It is noteworthy that no long-term results of this technique are available. Moreover, when evaluating the long-term results of sphincterplasty for patients with fecal incontinence, studies invariably describe a decrease in continence over the years.

In high FIA, there is little standardisation in sphincter preserving techniques, complicating interpretation of study results. In our enquiry, MAF was the most applied technique, followed by LIFT. Both strategies are well established and show no significant difference in overall healing and recurrence rate, as confirmed in a recent systematic review [16]. Incontinence rates were, however, significantly higher after MAF which might give LIFT a more favorable position in determining optimal procedure. It must be mentioned that owing to small numbers no separate analyses were performed concerning incontinence outcome in patients with cryptoglandular or Crohn’s FIA. Experience with MAF for high anal fistula was substantial which is in contrast to the survey by Ratto where surgeons were much less eager to perform a MAF, possibly due to its technically demanding character. Still, 8% of the participants treated high transsfincteric FIA with fistulotomy. The risk for impaired continence can be substantial [23]. When considering this approach in the individual patient it is advisable to carefully evaluate sphincter function and anatomy before surgery in order to estimate risk.

Almost 1/3(31%) of the respondents performed a fistulotomy in patients with an intersphincteric FIA with a high internal opening. This is in accordance with current guidelines [3, 5, 6] where this type of fistula is classified as ‘simple’ FIA. In an elegant study, incorporating pre- and postoperative sonography, Garcés-Albir et al. concluded that fistulotomy of the intersphincteric FIA, which involved less than 2/3 of the total length of the external anal sphincter, is a safe and effective treatment for patients without risk factors for fecal incontinence prior to surgery [41].

Experience with techniques other than MAF and LIFT was limited. Less than 10% of the respondents was familiar with more novel surgical approaches such as OTSC® and VAAFT. This was also the case for biomaterials and tissue-adhesive techniques like the anal fistula plug, fibrin glue and Permacol®. With 21% in this study compared to 10% in the study by Ratto [21] the FiLaC™ seems to be the most popular of these, although evidence of superiority of this procedure is not convincing [42, 43]. At the present time, it would seem prudent not to apply untested methods in our patients outside of trials or adequate prospective registries. Moreover, in our opinion, companies offering such technology should insist on only applying their new techniques within prospective registry.

FIA recurrence is significantly associated with an undetected internal fistula opening [44, 45]. Ninety-six percent of the respondents who did not find an internal opening proceeded to curettage of the fistula tract.

In the original description of the LIFT technique, the intersphincteric tract is sutured twice, namely at the point where it passes the internal and external anal sphincter. The internal orifice is left open. Of the respondents**,** 23% closed the internal opening, even though this was not described in the original LIFT technique [46]. Applying a procedural variation with the intention of improving results is understandable. However, it makes comparing results of fistula surgery difficult. A database exactly describing the procedure performed and patient characteristics would be of great help to evaluate results and determine best outcome instead of developing more and more procedures based on the same underlying mechanism of the origin of the FIA.

The strength of the present study was the response rate with 43%of respondents also considering the fact that the survey invitation was not individualized [47]. The subject of the study is partially responsible for the high response rate since it was of great interest to most respondents. Forty-seven percent of the respondents were members of the Dutch Coloproctology Working group, a well-known coloproctology society in the Netherlands.

Some limitations of the study should be mentioned. The most important one is probably the classification of fistula which is, to a certain extent, surgeon dependent. This might have caused confusion when answering the questions and might have influenced our results. Another limitation may be the personal interpretation of the answer options resulting in intrinsic selection bias. The questionnaire was sent to all members of the Dutch Coloproctology Working group. Its members are all practicing and interested in colorectal disease but also include residents besides gastrointestinal surgeons. Efforts were made to send the survey to all known surgeons who were not members of the Workgroup but still known to be familiar with anorectal disease. This was done by calling the secretariat of each hospital. Nevertheless, it is likely that not all surgeons were reached. Software related issues could also have jeopardized the response rate because personalized correspondence was not possible.

In summary, this study shows consistency in the treatment of low FIA between respondents, whereas in high FIA treatment is more variable. The results also suggest that there is a lack of consensus regarding performing diagnostic imaging, seton placement and how to manage the internal fistula opening.

## Conclusions

Varying practices are seen among gastrointestinal surgeons concerning the management of FIA and a considerable part of the respondents appear to treat FIA differently than recommended in guidelines. Novel promising techniques should be investigated adequately in sufficiently large trials and in prospective registries to increase consensus. The development of a Core Outcome Set for FIA may improve the quality and uniformity of future research. Treatment should be patient tailored with meticulous assessment of fistula characteristics prior to surgery to obtain the best results, but with a consistent practice of laying open low FIA and sphincter-preserving techniques for high transsphincteric FIA.

## Appendix

### Personal data

1. You are a
  - Colorectal surgeon
  - General surgeon
  - Fellow
  - Surgical resident (in training)
  - Other
2. Gender
  - Male
  - Female
3. Do you work?
  - Fulltime
  - Part-time
4. Where do you work?
  - Academic hospital
  - Non-academic hospital
  - (Private) clinic
5. When a patient with FIA visits the outpatient clinic, he is seen by *(mc)*
  - The specialist
  - The fellow
  - The surgical resident in training
  - The surgical resident not in training
  - The nurse practitioner or physician assistant
6. Personal experience with surgical management of FIA?
  - 1–5 years
  - 5–10 years
  - 10–20 years
  -  > 20 years
7. How many surgical procedures do you perform per year?
  - 0–10
  - 10–30
  - 30–50
  -  > 50

### Diagnostic technique

8. When do you use diagnostic imagine? (mc)
  - Recurrent FIA
  - Complex FIA
  - Prior to seton placement
  - Prior to surgical procedure
  - Prior to abscess drainage
  - Always
9. What diagnostic technique do you use? *(mc)*
  - MRI
  - CT
  - Endo-anal ultrasound
  - No diagnostic technique at all

### Seton treatment

10. When do you use seton placement? *(mc)*
  - Always
  - Purulent FIA
  - High FIA
  - Recurrent FIA
  - Never
11. What type of seton do you use? *(mc)*
  - Silicone (e.g., vessel loop)
  - Comfort drain
  - Surgical thread (e.g., mersilene)
  - SuperSeton®
12. What moment do you remove the seton?
  -  < 6 weeks
  - Between 6 weeks and 3 months
  -  > 3 months
  - Till next surgical procedure

### Surgical techniques

13. Which surgical treatment do you perform in a patient with a low transsphincteric FIA? *(mc)*
  - Fistulotomy
  - Mucasal advancement flap (MAF)
  - Ligation of the intersphincteric fistula tract (LIFT)
  - Plug
  - Permacol paste
  - Laser (FiLaC™)
  - Fibrin glue
  - Video-assisted anal fistula treatment (VAAFT)
  - Over-the-scope-clip (OTSC®)
  - With direct sphincter repair if necessary
14. Which surgical treatment do you perform in a patient with a high transsphincteric FIA? *(mc)*
  - Fistulotomy
  - Mucasal advancement flap (MAF)
  - Ligation of the intersphincteric fistula tract (LIFT)
  - Plug
  - Permacol paste
  - Laser (FiLaC™)
  - Fibrin glue
  - Video-assisted anal fistula treatment (VAAFT)
  - Over-the-scope-clip (OTSC®)
  - With direct sphincter repair if necessary
15. Which surgical treatment do you perform in a patient with a low intersphincteric FIA? *(mc)*
  - Fistulotomy
  - Mucasal advancement flap (MAF)
  - Ligation of the intersphincteric fistula tract (LIFT)
  - Plug
  - Permacol paste
  - Laser (FiLaC™)
  - Fibrin glue
  - Video-assisted anal fistula treatment (VAAFT)
  - Over-the-scope-clip (OTSC®)
  - With direct sphincter repair if necessary
16. Which surgical treatment do you perform in a patient with a high intersphincteric FIA? *(mc)*
  - Fistulotomy
  - Mucasal advancement flap (MAF)
  - Ligation of the intersphincteric fistula tract (LIFT)
  - Plug
  - Permacol® paste
  - Laser (FiLaC™)
  - Fibrin glue
  - Video-assisted anal fistula treatment (VAAFT)
  - Over-the-scope-clip (OTSC®)
  - With direct sphincter repair if necessary

### Experience surgical approaches

17. What is your experience with the MAF?
  - No experience
  - 1–5 years
  - 5–10 years
  - 10–20 years
  -  > 20 years
18. What is your experience with the LIFT procedure?
  - No experience
  - 1–5 years
  - 5–10 years
  - 10–20 years
  -  > 20 years
19. What is your experience with treatment with a plug?
  - No experience
  - 1–5 years
  - 5–10 years
  - 10–20 years
  -  > 20 years
20. What is your experience with Permacol® paste?
  - No experience
  - 1–5 years
  - 5–10 years
  - 10–20 years
  -  > 20 years
21. What is your experience with the laser (FiLaC™)?
  - No experience
  - 1–5 years
  - 5–10 years
  - 10–20 years
  -  > 20 years
22. What is your experience with fibrin glue?
  - No experience
  - 1–5 years
  - 5–10 years
  - 10–20 years
  -  > 20 years
23. What is your experience with the video-assisted anal fistula treatment (VAAFT)?
  - No experience
  - 1–5 years
  - 5–10 years
  - 10–20 years
  -  > 20 years
24. What is your experience with the Over-the-scope-clip (OTSC®)
  - No experience
  - 1–5 years
  - 5–10 years
  - 10–20 years
  -  > 20 years

### Internal opening

25. When do you close the internal opening? *(mc)*
  - Always
  - Never
  - When performing a MAF
  - When performing a LIFT
  - When performing a plug
  - When performing Permacol® paste
  - When performing laser (FiLaC™)
  - When performing fibrin glue
  - When performing VAAFT
  - When performing (OTSC®)
26. How do you close the internal opening?
  - Z-suture
  - Normal suture
  - Not applicable, I do not close the internal opening
  - Otherwise, namely..
27. What if you do not find an internal opening?
  - Only fistulectomy or core out of the fistula tract
  - Excoriation of the fistula tract
  - I do nothing
28. How do you perform the excoriation?
  - With a curette
  - With a brush
  - With a gauze
  - Otherwise, namely.
